# Multi-omics analysis revealed TEK and AXIN2 are potential biomarkers in multifocal papillary thyroid cancer

**DOI:** 10.1186/s12935-022-02606-x

**Published:** 2022-05-12

**Authors:** Ga Hyun Kim, Hye Jin Heo, Ji Wan Kang, Eun-Kyung Kim, Seung Eun Baek, Keunyoung Kim, In Joo Kim, Sunghwan Suh, Byung-Joo Lee, Yun Hak Kim, Kyoungjune Pak

**Affiliations:** 1 Interdisciplinary Program of Genomic Data Science, Pusan National University, Yangsan, Republic of Korea; 2 Department of Anatomy, School of Medicine, Pusan National University, Yangsan, Republic of Korea; 3 Department of Nuclear Medicine and Biomedical Research Institute, Pusan National University Hospital, Busan, Republic of Korea; 4 Department of Internal Medicine, Dong-A University College of Medicine, Busan, Republic of Korea; 5 Department of Otorhinolaryngology-Head and Neck Surgery, Pusan National University Hospital, Busan, Republic of Korea; 6 Department of Biomedical Informatics, School of Medicine, Pusan National University, Yangsan, Republic of Korea

**Keywords:** Papillary thyroid carcinoma, BRAF mutation, Multifocality, Multi-omics

## Abstract

**Background:**

Papillary thyroid carcinoma (PTC), the most common endocrine cancer, accounts for 80–85% of all malignant thyroid tumors. This study focused on identifying targets that affect the multifocality of PTC. In a previous study, we determined 158 mRNAs related to multifocality in BRAF-mutated PTC using The Cancer Genome Atlas.

**Methods:**

We used multi-omics data (miRNAs and mRNAs) to identify the regulatory mechanisms of the investigated mRNAs. miRNA inhibitors were used to determine the relationship between mRNAs and miRNAs. We analyzed the target protein levels in patient sera using ELISA and immunohistochemical staining of patients’ tissues.

**Results:**

We identified 44 miRNAs that showed a negative correlation with mRNA expression. Using in vitro experiments, we identified four miRNAs that inhibit TEK and/or AXIN2 among the target mRNAs. We also showed that the downregulation of *TEK* and *AXIN2* decreased the proliferation and migration of BRAF ( +) PTC cells. To evaluate the diagnostic ability of multifocal PTC, we examined serum TEK or AXIN2 in unifocal and multifocal PTC patients using ELISA, and showed that the serum TEK in multifocal PTC patients was higher than that in the unifocal PTC patients. The immunohistochemical study showed higher TEK and AXIN2 expression in multifocal PTC than unifocal PTC.

**Conclusions:**

Both TEK and AXIN2 play a potential role in the multifocality of PTC, and serum TEK may be a diagnostic marker for multifocal PTC.

**Supplementary Information:**

The online version contains supplementary material available at 10.1186/s12935-022-02606-x.

## Introduction

Thyroid cancer is the most common malignant endocrine tumor and over the last three decades, its incidence has increased continuously worldwide [1, 2]. The most common form of thyroid cancer, papillary thyroid carcinoma (PTC), accounts for 80–85% of all malignant thyroid tumors [3], and has a favorable prognosis with excellent survival rates. However, a minority of patients with PTC develop locoregional recurrence, including cervical lymph node metastases, which eventually leads to mortality in some patients [4]. PTC often presents with multiple anatomically distinct foci within the thyroid, known as multifocal PTC. The reported prevalence of multifocal PTC ranges from 18 to 87% [5, 6]. However, it remains controversial whether multifocal PTCs are (1) multiple synchronous independent primary tumors or (2) intraglandular dissemination of a single common malignant clone [7, 8]. Previously, we identified the overexpression of 158 mRNAs in multifocal BRAF ( +) PTCs, compared to unifocal BRAF ( +) PTCs [9]. However, none of the mRNAs in BRAF ( +) PTCs showed lower expression than that of the mRNAs in unifocal PTCs [9]. Additionally, none of the mRNAs showed significantly different expression between multifocal and unifocal BRAF (−) PTCs [9]. In addition, mRNAs that were overexpressed in multifocal BRAF ( +) PTCs were associated with Wnt- and pluripotency-related pathways, which might account for the difference between multifocal and unifocal BRAF ( +) PTCs [9]. The multifocality of PTC has a clinical impact on the treatment of PTC. Although multifocality was not considered as a risk factor in the American Joint Committee on Cancer/Union for International Cancer Control (AJCC/UICC) staging system [4] or American Thyroid Association (ATA) guidelines [4], multifocal PTC has an increased risk of lymph node metastasis, recurrence, and distant metastasis [10, 11]. In addition, multifocality of PTC is associated with the decision making on the optimal extent of surgery for PTCs, if it was identified before the surgery. However, fine needle aspiration cannot be performed for multiple thyroid nodules in clinical settings. The extent of surgical management affects the prognosis of PTC in terms of recurrence [12] and survival [13]. Therefore, adequate information regarding the multifocality of PTC should be provided to improve decision making.

MicroRNAs (miRNAs), a class of non-coding RNAs (15–27 nucleotide RNA molecules), control the expression of mRNA post-transcriptionally by binding to the 3ʹ-untranslated region of mRNA by blocking translation or mRNA degradation [14, 15]. As a large number of miRNAs have been discovered with the continuous progress of technology, the importance of miRNA-mRNA regulatory mechanisms in physiological and pathological states is slowly becoming highlighted [14, 16]. Recently, studies of miRNAs have focused mostly on malignant neoplasms, and miRNAs have been shown to play pivotal roles in various cancers by regulating the expression of their target mRNAs [17–19].

In this study, we hypothesized that miRNAs interact with overexpressed mRNAs in multifocal BRAF ( +) PTCs, regulating protein expression. Therefore, we investigated target miRNAs by exploring the following: (1) screening miRNAs that interact with mRNAs in multifocal BRAF ( +) PTCs, (2) validation of miRNAs with functional assays, and (3) protein expression in blood samples from patients with PTCs.

## Materials and methods

### Data acquisition

Clinical characteristics and gene expression data (mRNAs and miRNAs) for PTC were downloaded from the Genomic Data Commons Data Portal (https://gdc-portal.nci.nih.gov/). The Cancer Genome Atlas (TCGA) data were available without restrictions on publications or presentations according to TCGA publication guidelines. Patients were categorized according to BRAF mutation status. In addition, patients were divided into two groups according to the multifocality of PTC. Of the 237 patients with BRAF ( +) PTCs, 110 had multifocal PTC and 127 had unifocal PTC according to our previous research [9].

### Cell culture

BCPAP (PTC cell line) harboring the BRAF mutation was purchased from DSMZ Korea and was maintained in Roswell Park Memorial Institute (RPMI) 1640 medium containing 10% fetal bovine serum (FBS) and 100 µg/mL penicillin–streptomycin. All cultures were incubated at 37 °C in the presence of 5% CO_2_.

### microRNA (miRNA) inhibitor transfection

BCPAP cells were transfected with hsa-miR-21a-5p mirVana® miRNA inhibitor (#4,464,084, ID: MH12979, Thermo Fisher Scientific, Waltham, MA, USA), hsa-miR-34a-3p mirVana® miRNA inhibitor (#4,464,084, ID: MH13089, Thermo Fisher Scientific), hsa-miR-203a-3p mirVana^®^ miRNA inhibitor (#4,464,084, ID: MH10152, Thermo Fisher Scientific), hsa-miR-362-3p mirVana^®^ miRNA inhibitor (#4,464,084, ID: MH12485, Thermo Fisher Scientific) and negative control (#4,464,076; Thermo Fisher Scientific). These were used at a final concentration of 30 nM and transfected using Lipofectamine RNAiMAX reagent (Thermo Fisher Scientific).

### miRNA assay

For assessment of miRNA expression changes, RT-PCR was performed after miRNA inhibitor transfection. Primers for hsa-miR-21a-5p (#A25576, ID: 477,973 mir), hsa-miR-34a-3p (#A25576, ID: 478,047 mir), hsa-miR-203a-3p (#A25576, ID: 478,316 mir), hsa-miR-362-3p (#A25576, ID: 478,058 mir) was purchased from Thermo Fisher Scientific. miRNA was extracted using miRNeasy Mini Kit (Qiagen, Germany) and reverse transcription was conducted using a TaqMan Advanced miRNA cDNA Synthesis Kit (Thermo Fisher Scientific). Real time-PCR was performed using the LightCycler TM system (Roche Applied Science, Indianapolis, IN, USA).

### siRNA transfection

Negative control (Bioneer, South Korea, #SN-1003) siRNA and target gene siRNA were purchased from Bioneer Corporation (Daejeon, South Korea). Sequences are in Table 1 To create the knockdown cell line, BCPAP cells were seeded at 8 × 10^4^ cells per well in a 6-well plate in RPMI containing 10% FBS. The cells were transfected using DharmaFECT 1 (Thermo Fisher Scientific), as per the manufacturer’s instructions, with 300 nM siRNA and incubated for 48 h.

**Table 1:** Sequence of siRNAs

siRNA	Forward(5ʹ-3ʹ)	Reverse(5ʹ-3ʹ)	Target gene
siTEK	UGAUGAGGUGUAUGAUCUA	UAGAUCAUACACCUCAUCA	TEK
siAXIN2	GACCACAGCCAUUCAGGAA	UUCCUGAAUGGCUGUGGUC	AXIN2
siADAMTS9	CAGGUUACACAACCCAACA	UGUUGGGUUGUGUAACCUG	ADAMTS9
siADAMTSL2	CUCUGUACCCGGAUGACU	UAGUCAUCCGGGUACAGA	ADAMTL2

### Cell proliferation assay

Cells were seeded at a density of 5 × 10^3^ cells per well in 96-well plates in RPMI containing 10% FBS. After transfection with siRNA for 72 h, BCPAP cell viability was measured using the Cyto X cell viability assay kit from LPS solution Corporation (South Korea). Cyto X (10 µL) was added to each well and incubated for 1 h in a CO_2_ incubator. Optical density (OD) values were quantitatively measured at 450 nm using an enzyme-linked immunosorbent assay reader.

### Real-time PCR

Total RNA was extracted using the RNeasy mini kit (Qiagen, Germany), miRNA was extracted using miRNeasy mini kit (Qiagen), and cDNA synthesis was performed using a cDNA synthesis kit (Smart gene, South Korea, # SG-CDNAC100). Real-time monitoring of PCR reactions was performed using the LightCycler TM system (Roche Applied Science) and SYBR Green Q-PCR Master Mix with Low Rox (Smart gene, South Korea, #SG-SYBR-ROXL). Glyceraldehyde-3-phosphate dehydrogenase (GAPDH) was used as an internal control and primer sequences for real-time PCR (Table 2).

**Table 2:** List of RT-PCR primers

Gene	Forward(5ʹ-3ʹ)	Reverse(5ʹ-3ʹ)
ABCB1	CATTCAGGTTTCATTTTGGTG	TCCAACCACGTGTAAATCCTA
ADAMTS9	CATGCAGTTTGTATCCTG	GCGTTCTTTTGAAGTGGACG
ADAMTSL2	ATGTCCACATCTCCAGCAAAC	AGAGTAACCAAGGTGGGCATT
AGPAT5	ACGAGAAAGAGATGCGAAACA	AAGCAACGTGAGTTGCCTTTA
ARAP3	TGGTTGCCTGTACTATGGTGT	GTGAAATGAGGTCATCACTGG
AXIN2	CTTATCGTGTGGGCAGTAAGA	TCTCTTCATCCTCTCGGATCT
BCAS3	CTGAAGCCAAAGTACAGGACA	CTCATGCGATTCACTACTCGT
BMPR2	TGAAACAAGTCGAAACTGGAG	ATTAATATTCAGCCGGGTGTC
BOC	TCAGTGTACGTGACCTGGATT	TTGTAGGAGGTGCCTTTCTCT
B4GALT6	CAGACCAGAGGGAGACTTAGG	CTCTGGCATGAGGTTTACAGA
CCND2	GTGCATTTACACCGACAACTC	CACACAGAGCAATGAAGGTCT
CD44	CAAATCATTCTGAAGGCTCAA	GGGTGTCCTTATAGGACCAGA
ELOVL2	GCACAAGTATCTTTGGTGGAA	AGGTGGCTCTTGCATATCTTT
ETS1	TGCAGAAAGAGGATGTGAAAC	ATTCTGCAAGGTGTCTGTCTG
FZD3	TTTTTACTATGGCTGGCAGTG	ATCTCAATGCATCAACATCGT
FZD4	AGAGAGTCTGAACTGCAGCAA	GTACTGTGAAGGCAGTGGAGA
HEY2	CGGGATCGGATAAATAACAGT	TAGGCACTCTCGGAATCCTAT
HIST1H2AC	GTCTGGACGTGGTAAGCAAG	ATGATGCGAGTCTTCTTGTTG
HIST1H4H	GTAAAGGTGGAAAAGGTTTGG	CGTGCTCTGTGTAAGTGACAG
PDGFD	CTAGATTCCCGAACAGCTACC	TCATCGGACTTGAATGTGATT
PIK3R3	TAAATGACAAATTGCGGGATA	TGGGATTGTACTGAGCAAGAG
PODXL	CTCAACAGACCTCCAGTCAGA	CACTTTGCCCAGTTACTCTCA
RALB	GAACAGATTCTCCGTGTGAAG	TTCTTGCCATTCTTGTCTTTG
RAPGEF4	CAGCCTTCACAAGGTACAGAA	ATCTTCTCCAACTCTGGCAGT
RNF146	CAAAGAAGGGAGTAGCTGGAC	TCTTCTCCTTCTCCCCTATGA
SLIT3	ACGCCTAGAACAGAACTCCAT	CATACAGGGAGAGCAAGTTGA
TCF4	AGTAAAACAGAAAGGGGCTCA	GCATAGACTGAAGATGGCAAA
TEK	TAAACTTGGACACCATCCAAA	CCAGATCCCTGTGGATAAACT
THSD7B	CCTGCTAAGACCATCACTGAA	TCTCCAAATTATGCTGCTCAC
TRPC4	TTTCCTGTCTTCTCTGTGTGC	CACGGTAATATCATCCACTCG

### Wound healing assay

In order to measure the cell migration during wound healing, 48 h after the transfection was carried out (in a 100 mm cell culture dish), BCPAP cells were replated in 24-well plates at a density of 3 × 10^5^ cells per well. Twenty-four hours later, BCPAP cells were cultured in RPMI containing 10% FBS and treated with 1 µg/mL mitomycin-C (Sigma-Aldrich, USA) for 3 h and then wounded with a linear scratch using SPLScar™ Scratcher (SPL Science, South Korea). The average extent of the wound area was evaluated by measuring the width of the wound using ImageJ software.

### Three-dimensional (3D) spheroid formation assay

3D spheroid formation was examined by culturing the cells in 200 μL complete medium containing 300 cells in each well and cultured in an ultra-low attachment 96-well plate (Corning, USA, #7007). After 1, 3, and 5 days of incubation, spheroid formation was photographed using phase contrast microscopy (4 × magnification).

### Western blot

Cells were washed with PBS, dissolved in radioimmunoprecipitation assay (RIPA) buffer supplemented, and centrifuged at 15,000×*g* for 10 min at 4 °C. Protein concentration was determined by the BCA protein assay (#23,227, Thermo Fisher Scientific) using bovine serum albumin (BSA) as the standard. The proteins were separated by SDS–PAGE and transferred to hybridization nitrocellulose filter membranes (Merck Millipore, USA). The membranes were blocked for non-specific binding with 5% BSA in Tris-buffered saline containing TBS-T (TBS with 0.1% Tween 20) for 1 h at room temperature and then incubated with specific primary antibodies (diluted 1:1000) in TBS-T at 4 °C overnight. After washing with TBS-T three times, the proteins were identified using appropriate secondary antibodies (diluted 1:2000 with 5% BSA). Chemiluminescence was detected with SuperSignal™ West Dura Extended Duration Substrate (Thermo Scientific, USA, #34,075) and visualized using an Amersham Imager 680 (GE Healthcare, USA).

### Blood samples

Blood samples were collected from 90 patients with BRAF ( +) PTCs (42 unifocal PTCs and 48 multifocal PTCs) who underwent total thyroidectomy at Pusan National University Hospital. Samples were collected within one week of surgery. TEK (Mybiosource, USA, #MBS175906), AXIN2 (Mybiosource, USA, #MBS046455), ADAMTS9 (Novusbio, USA. #NBP2-66,447) levels in serum were evaluated using an ELISA kit according to the manufacturer’s instructions. The results were recorded and analyzed using a microplate reader at 450 nm wavelength (TECAN, Switzerland).

### Immunohistochemistry

Human tumor tissue paraffin blocks were processed into sections and deparaffinized, followed by incubation with 3% hydrogen peroxide for 20 min to block the endogenous peroxidases. Next, the tissue sections were blocked with 1% bovine serum albumin for 30 min and then incubated with a polyclonal antibody against AXIN2 and TEK (ABclonal Technology; 1:100 dilution) for overnight at 4 °C. Immunoreaction was visualized using the EnVision detection system kit (Dako), and Mayer's hematoxylin solution was used to stain the nuclei. After staining, images were obtained using an Axio Scan Z1 Digital Slide Scanner (Zeiss, Germany) and analyzed using Zen Blue software (Carl Zeiss. Germany) (magnification: × 100).

### Statistical analysis

Statistical differences in clinical variables were analyzed using the chi-square test. To determine the relationship between the 145 mRNAs and total miRNAs, we used the Spearman correlation method based on the Hmisc R package (Hmisc version 4.0–3 and R version 3.4.3). From the correlation analysis results, we selected targets with negative correlations of 0.1 or more, and searched the genes related to the selected miRNAs in Tarbase v.8 (http://carolina.imis.athena-innovation.gr/diana_tools/web/index.php?r=tarbasev8) to confirm the correlation. We sorted the selected miRNAs by 13 pathways identified in previous studies. In vitro experimental data were statistical significance of the differences among groups was determined by a one‐way analysis of variance (ANOVA) followed by Tukey's test for multiple comparison using the GraphPad Prism 5 software (GraphPad Software, La Jolla, CA) and differences were considered statistically significant at p < 0.05. A comparison of receiver operating characteristic (ROC) was performed to test the difference between the area under the curve of two ROCs from blood samples using MedCalc 19.8 (MedCalc Software Ltd, Ostend, Belgium).

## Results

### Screening of potential regulatory miRNAs of 145 mRNAs related to multifocality

**Fig. 1:**
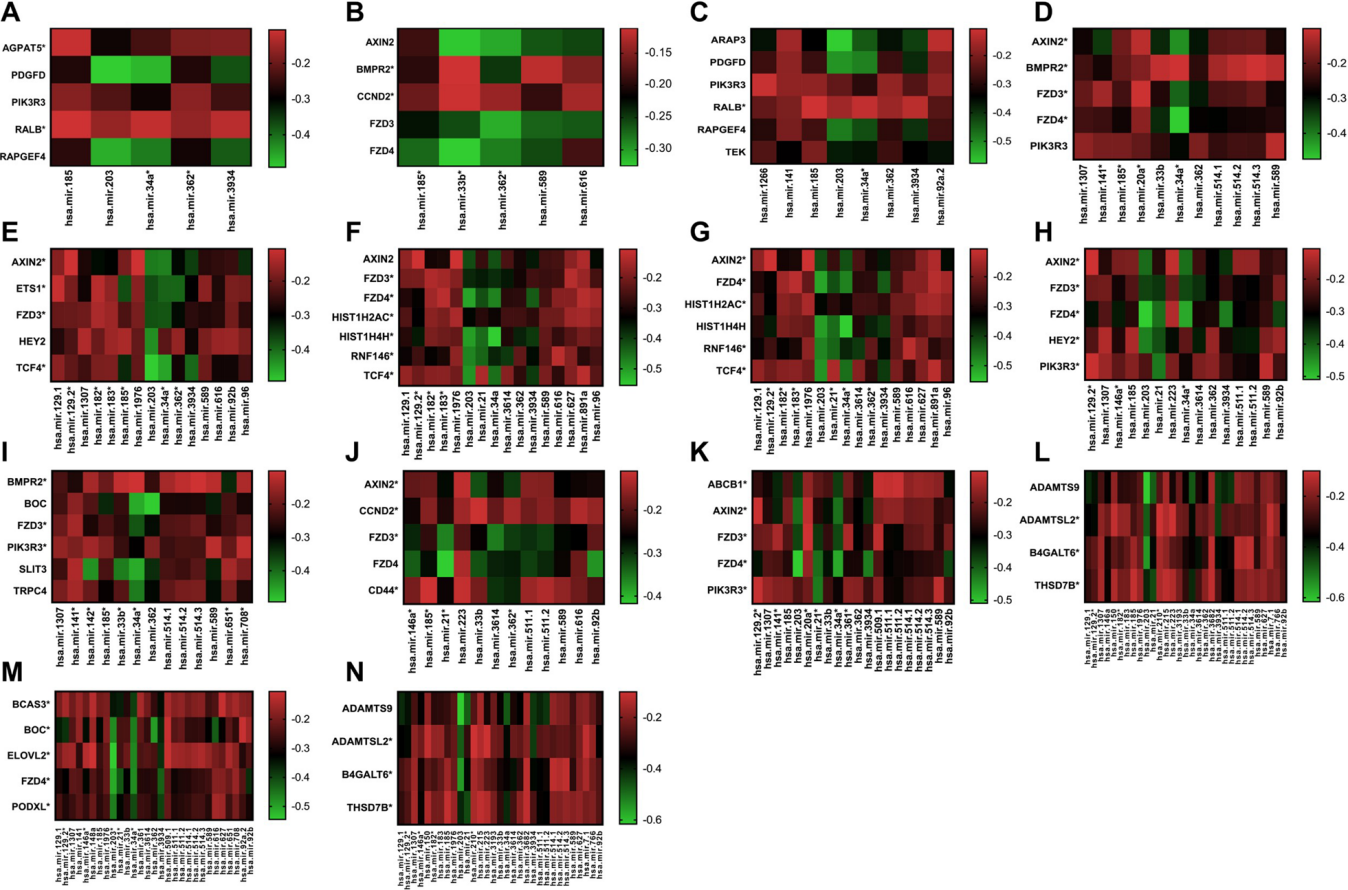
The correlation between target mRNAs in each pathway and miRNAs in TCGA. **A** Phospholipase D signaling pathways. **B** Hippo signaling pathway. **C** Rap1 signaling pathway. **D** Signaling pathways regulating pluripotency of stem cells. **E** Neural crest differentiation. **F** Signaling by Wnt (**G**) TCF dependent signaling in response to Wnt. **H** Breast cancer. **I** Axon guidance. **J** Wnt signaling pathway and pluripotency. **K** Gastric cancer. **L** Ectoderm differentiation (**M**) O-linked glycosylation. (The * means it is a known target in the Tarbase)

In the previous study, we reported the enriched genes and pathways in multifocal PTC compared to unifocal PTC [9]. Since multifocal PTC is a more aggressive cancer, we focused on the functional role and regulatory mechanisms of the genes based on their pathways. To obtain more convincing potential regulatory mechanisms, we used multi-omics data from TCGA. 145 mRNAs in the enriched pathways and whole miRNAs were included in the correlation analysis. miRNAs that correlated negatively with mRNAs were discovered in each pathway as follows [9]: 13 miRNAs in axon guidance, 15 in breast cancer, 29 in ectoderm differentiation, 20 in gastric cancer, 5 in the Hippo signaling pathway, 15 in neural crest differentiation, 31 in O‐linked glycosylation, 5 in Phospholipase D signaling pathway, 8 in Rap1 signaling pathway, 16 in Wnt signaling pathway, 11 in signaling pathways regulating pluripotency of stem cells, 16 in TCF dependent signaling in response to Wnt, 12 in the Wnt signaling pathway [20–41] (Fig. 1). To select highly relevant genes, the relationship between mRNAs and miRNAs was confirmed by using Tarbase (Table 3 and Additional file 3: Table S1). Combining the results of Tarbase and correlation analysis, we selected four miRNAs (miR21, miR34a, miR203, and miR362) as potential regulators of target mRNAs.

**Table 3:** The result of correlation analysis. Only values with a correlation coefficient < − 0.1 and P-value < 0.05 were indicated

Gene symbol	miR203 correlation coefficient	miR203 P-value	miR21 correlation coefficient	miR21 P-value	miR34a correlation coefficient	miR34a P-value	miR362 correlation coefficient	miR362 P-value
ABCB1	–	–	–	–	− 0.368	4.61E−09	–	–
ADAMTS9	− 0.616	0	− 0.476	6.66E−15	–	–	–	–
ADAMTSL2	− 0.475	7.77E−15	− 0.295	4.00E−06	–	–	–	–
AXIN2	− 0.414	2.62E−11	− 0.271	2.10E−05	− 0.418	1.60E−11	− 0.306	1.00E−06
B4GALT6	− 0.55	0	–	–	–	–	–	–
BCAS3	− 0.348	3.24E−08	–	–	–	–	–	–
BMPR2	–	–	–	–	–	–	− 0.247	0.000117
BOC	− 0.474	8.44E−15	–	–	–	–	–	–
CCND2	–	–	− 0.241	0.000171	–	–	− 0.139	0.0312
ELOVL2	− 0.545	0	–	–	–	–	–	–
ETS1	− 0.371	3.43E−09	–	–	–	–	–	–
FZD3	–	–	–	–	–	–	− 0.309	1.00E−06
HEY2	− 0.435	1.98E−12	–	–	–	–	–	–
HIST1H2AC	− 0.337	9.20E−08	–	–	–	–	–	–
HIST1H4H	− 0.504	0	–	–	–	–	–	–
PIK3R3	− 0.232	0.000301	–	–	–	–	–	–
PODXL	− 0.461	5.93E−14	–	–	–	–	–	–
RNF146	− 0.476	6.88E−15	–	–	–	–	–	–
TEK	–	–	–	–	− 0.363	7.57E−09	–	–
THSD7B	− 0.406	6.54E−11	− 0.453	1.76E−13	–	–	–	–

### Selection of target genes through miRNA inhibitors treatment

**Fig. 2:**
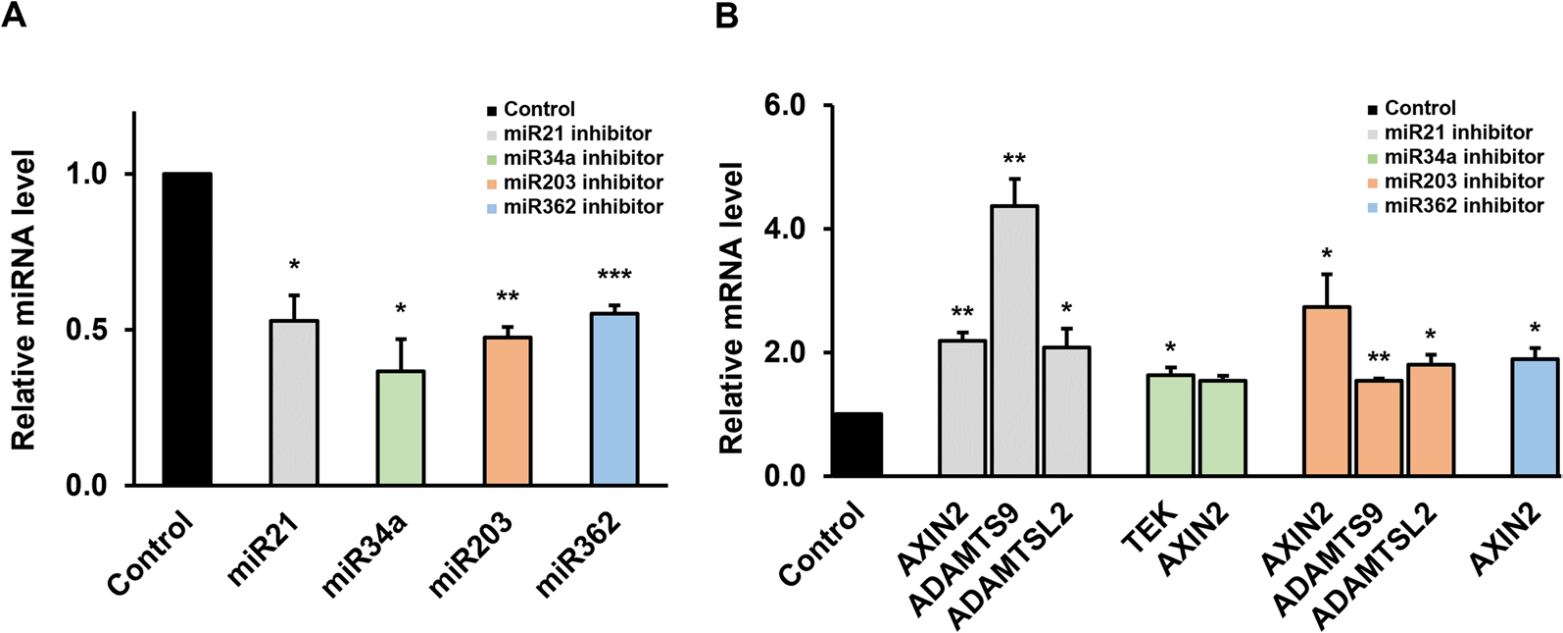
Inhibition of miR21, miR34a, miR203, and miR362 in BCPAP cells. **A** miRNA inhibitor transfection reduced the expression levels of all four miRNAs by more than 40% in BCPAP as analyzed by qPCR. **B** AXIN2, ADAMTS9 and ADAMTSL2 mRNA expression was up-regulated by miR21 inhibitor (gray), TEK, AXIN2 mRNA expression was up-regulated by miR34a inhibitor (green). And AXIN2, ADAMTS9 and ADAMTSL2 mRNA expression was up-regulated by miR203 inhibitor (orange), AXIN2 mRNA expression was up-regulated by miR362 inhibitor (blue). *P < 0.05, **P < 0.01, ***P < 0.001, compared to the control

To validate the relationship between target mRNAs and miRNAs, BCPAP cells were treated with inhibitors of miR21, miR34a, miR203, and miR362. The expression of each miRNA was suppressed after 24 h (Fig. 2A). Four upregulated genes (*TEK*, *AXIN2*, *ADAMTS9*, and *ADAMTSL2*) were shown to be miRNA-regulated genes after treatment with miRNA inhibitors (Fig. 2B). Negative results are presented in Additional file 1: Figure S1.

### Functional assays of target mRNAs through siRNA treatment

**Fig. 3:**
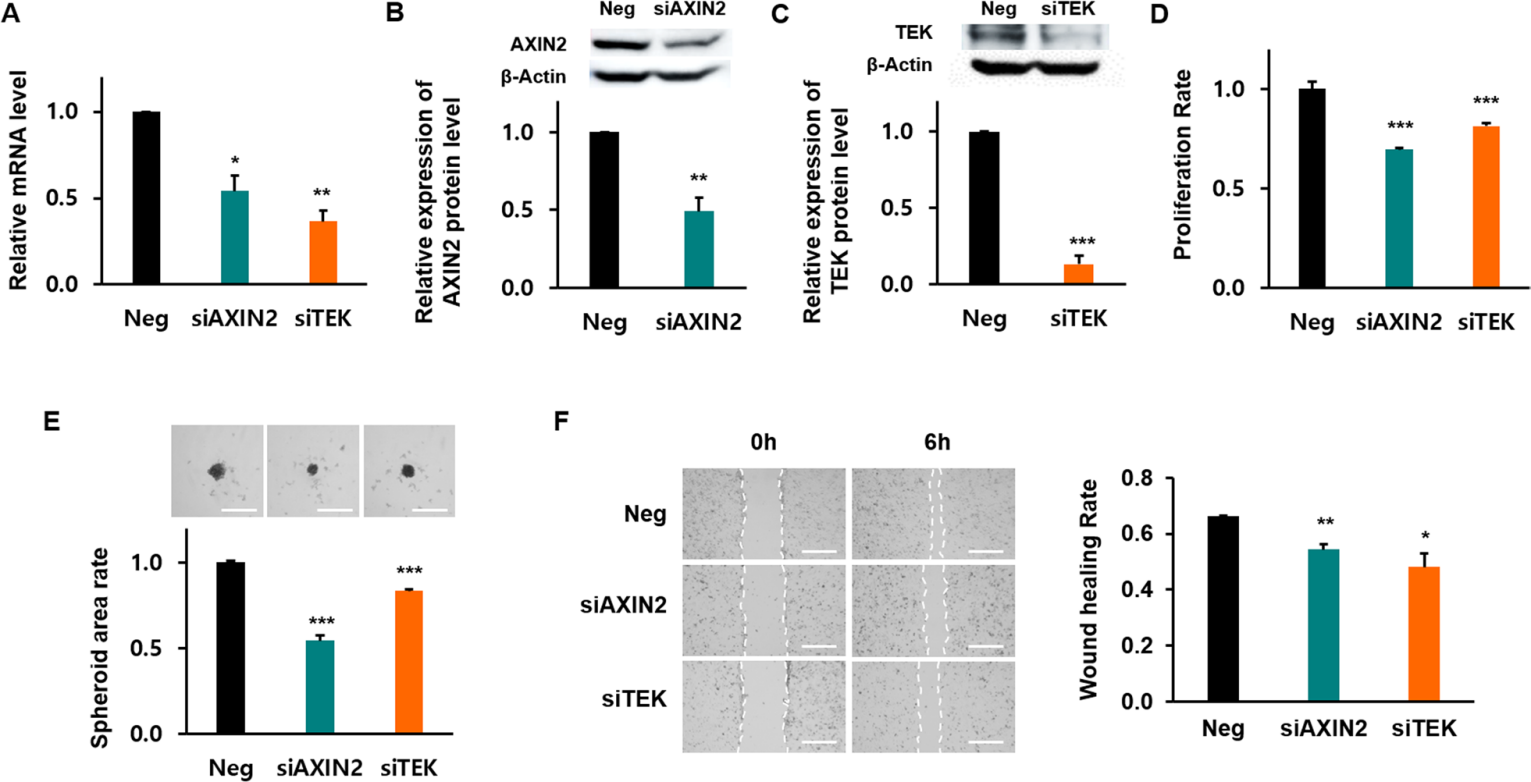
AXIN2, TEK knockdown inhibits the proliferation, migration of BRAF ( +) BCPAP cells. **A** AXIN2, TEK siRNA decreased AXIN2, TEK mRNA expression in BCPAP cells (n = 3). **B**, **C** Western blotting analysis of AXIN2, TEK knockdown efficiency in BCPAP cells. **D** Cell proliferation assay showed that AXIN2, TEK siRNA suppressed the proliferation of BCPAP cells. **E** After transfection with AXIN2, TEK siRNA or control siRNA, spheroid formation was evaluated at 3 days post-seeding. The spheroid area was measured using ImageJ software, encompassing the entire spheroid to the outside edge of spheroid. Scale bar indicates 1000 µm. **F** Wound healing assay showed that AXIN2, TEK knockdown resulted in lower migration capacity in BCPAP cells than in negative control cells. Scale bar indicates 1000 µm. (n = 4) Values are means ± SEM *P < 0.05, **P < 0.01, ***P < 0.001

The knockdown efficiency was assessed by RT-PCR and western blotting, and the results showed that *TEK* and *AXIN2* expression was knocked down effectively by siRNA in BCPAP cells (Fig. 3A, B and C, and Additional file 2). To study whether this downregulation could inhibit the proliferation of BCPAP cells, their proliferation was monitored. As shown in Fig. 3D, the proliferation rate had significantly decreased on day 3 in siRNA-transfected cells compared to the negative control. In the 3D spheroid formation assay, the BCPAP cells were transfected prior to the generation of spheroids, which were then allowed to grow for 3 days. After 3 days of treatment with *TEK* and *AXIN2* siRNA, the diameter of 3D spheroids was smaller than that of the negative control group, indicating that the *TEK* and *AXIN2* siRNA complexes could significantly inhibit cell growth (Fig. 3E).

To evaluate whether blocking *TEK* and *AXIN2* gene expression affects the oncogenic behavior of BCPAP cells, we performed a cell migration assay. In a wound-healing assay, the artificial wound gap in plates of the negative siRNA-transfected BCPAP cells was significantly narrower than that of the *TEK*-and *AXIN2* siRNA-transfected BCPAP cells at 6 h (Fig. 3F). *ADAMTS9* and *ADAMTSL2* were excluded from the experiment because of the repeated heterogeneous results.

### TEK and AXIN2 from patients with BRAF ( +) PTCs: multifocal vs unifocal

**Fig. 4:**
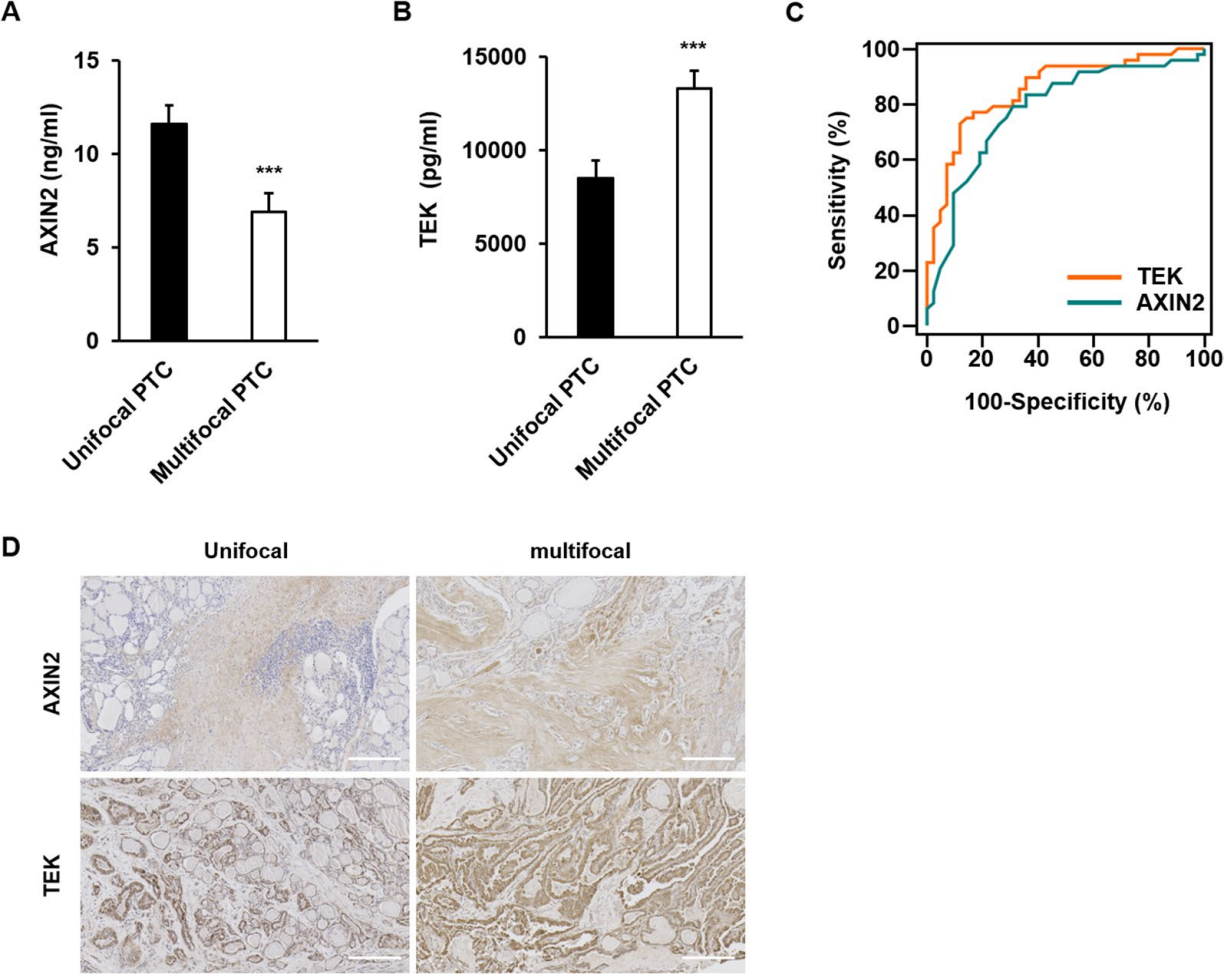
AXIN2, TEK were assayed using an ELISA and immunohistochemistry (Unifocal PTC, n = 42; Multifocal PTC, n = 48). The AXIN2 (**A**) and TEK (**B**) values in patients’ serum (median ± SEM). **C** The receiver operating characteristic (ROC) curve of TEK and AXIN2 in the PTC. **D** AXIN2 and TEK expression levels were confirmed by immunohistochemistry analysis in tumors (scale bar = 200 µm). Values are means ± SEM ***P < 0.001

42 patients with BRAF ( +) unifocal PTCs and 48 patients with BRAF ( +) multifocal PTC were included in this analysis. The sizes of the largest tumors were not significantly different (P = 0.5710) between unifocal (1.6 ± 0.7 cm) and multifocal (1.7 ± 0.8 cm) PTCs. The level of TEK in serum from multifocal PTCs was higher than that from unifocal PTCs (P < 0.0001, Fig. 4A), while the level of AXIN2 from multifocal PTCs was lower than that from unifocal PTCs (P < 0.0001, Fig. 4B). To test the performance of TEK and AXIN2, the areas under the curves were compared, which were not significantly different (TEK 0.854; AXIN2 0.779) (Fig. 4C). In the IHC results, the TEK and AXIN2 expression in multifocal PTC are much higher than unifocal PTC (Fig. 4D).

## Discussion

The results of the current study are summarized as follows: (1) 13 miRNAs interacted with mRNAs overexpressed in multifocal BRAF ( +) PTCs, (2) after validation with miRNA inhibitors, and functional assays, the mRNA expression of *TEK* and *AXIN2* was associated with the multifocality of BRAF ( +) PTCs, (3) TEK and AXIN2 in blood samples of patients with multifocal PTCs were significantly different from those with unifocal PTCs.

PTC is the most common form of thyroid cancer with a favorable prognosis; however, a minority of patients develop locoregional recurrence, which eventually leads to mortality in some of these patients [4]. Risk factors that affect the risk of recurrence in PTC include extrathyroidal extension, lymph node involvement, BRAF mutation status, tumor size, and sex [42]. Multifocal PTCs, defined as the presence of two or more than 2 anatomically separated tumor foci in the thyroid gland [43], have been associated with an increased risk of lymph node and distant metastases, as well as disease recurrence [44]. In clinical settings, physicians often encounter patients with multiple thyroid nodules; however, fine needle aspiration of multiple nodules is rarely performed. The selection of thyroid nodules for fine needle aspiration is mainly determined by the cancer probability from ultrasonographic findings, such as the content of the nodule, echogenicity, shape, margin, calcification, and vascularity [45]. According to the ATA guidelines [4], hemithyroidectomy is sufficient for unifocal PTCs without prior irradiation of the head and neck area, a history of familial thyroid cancer, and known lymph node metastasis. In contrast, total thyroidectomy can be recommended if the nodules are confirmed to be malignant in the bilateral lobes [46]. Therefore, decisions regarding the optimal extent of surgery for patients with multiple thyroid nodules should be made after careful consideration.

Based on the results of the correlation analysis of the TCGA data and the in vitro experiments, we identified the oncogenic role of *TEK* and *AXIN2* through miRNA regulatory mechanisms. Although we did not find the exact role of miRNAs in PTC, we observed that 4 miRNAs (miR21, miR34a, miR203, and miR363) inhibition increased the expression of *AXIN2* while *TEK* is only affected by mi34a in BRAF ( +) PTC cells. Along with the discovery of the candidate regulatory mechanisms, four miRNAs and two mRNAs were also validated in this study.

TEK (i.e., Tie2) is a receptor tyrosine kinase, which is mainly expressed in endothelial cells and controls vascular regeneration and stabilization [47]. The angiopoietin-Tie system is known to be involved in inflammation, metastasis, and lymphangiogenesis; therefore, multiple clinical trials were performed with selective Tie2 inhibitors [48]. However, the role of TEK in cancer cells remains unclear. Knockdown of TEK increased the proliferation and migration of clear cell renal cell carcinoma [49]; however, the overexpression of TEK in glioma cells was associated with tumor malignancy and drug resistance [50, 51]. Therefore, *TEK* in BRAF ( +) PTC cells may affect cell proliferation, invasion, and multifocality.

Since AXIN2 acts as an inhibitor of canonical Wnt signals in normal cells, many studies have focused on elucidating their role as tumor suppressors. However, silencing *AXIN2* decreases the invasive and metastatic characteristics of colon cancer [52]. In the current study, *AXIN2* expression was higher in multifocal PTC than in unifocal PTC, and knockdown of *AXIN2* inhibited cell proliferation in BRAF ( +) PTC cells. In addition, it was found that the expression of *Wnt* target genes, including AXIN2, differed based on the presence/absence of the BRAF mutation [53]. Our previous results showed that *AXIN2* expression did not differ between BRAF (−) multifocal and unifocal PTC [9]. Taken together, these results suggest that the role of *AXIN2* may be different for each cancer, and BRAF mutations may affect *AXIN2* expression.

In this study, the level of TEK in the serum from multifocal PTC patients was higher than that in unifocal PTCs, while the level of AXIN2 from multifocal PTCs was lower than that from unifocal PTCs. On the contrary, a lower level of AXIN2 was observed in multifocal PTC. The biological reason for this phenomenon may be the half-life of the protein [54, 55] as well as post-transcriptional and post-translational modifications. According to a recent analysis, protein expression correlates with the corresponding mRNA level by 20–40%, and mRNA expression levels are not completely representative of the corresponding protein concentration [56, 57]. Therefore, serum TEK and AXIN2 levels may provide information on multifocality in patients with BRAF ( +) PTC in a separate way.

Summarily, we used big database to obtain potential regulatory mechanism of target genes and highlighted the interaction of miRNAs with the expression of target genes and proteins in multifocal BRAF ( +) PTC. miRNA inhibition increased the mRNA expression of *TEK* and *AXIN2*. Serum TEK and AXIN2 levels may provide information on the multifocality of BRAF ( +) PTC. Although there are some limitations, this study provides evidences for the regulatory mechanism of the genes and their contribution to PTC multifocality for the first time.

## Supplementary Information

**Additional file 1:** **Figure S1.** Expression changes of mRNAs in BRAF (+) BCPAP cells after treatment with miR21 (gray), miR34a (green), miR203 (orange), and miR362 (blue) inhibitors at 24hr.

**Additional file 2:** **Figure S2.** Raw Western blot data.

**Additional file 3:** **Table S1.** The results of Tarbase.

## Data Availability

The datasets used or analysed during the current study are available from the corresponding author on reasonable request.
