# Supplementary figures and images for: Multi-omics analysis revealed TEK and AXIN2 are potential biomarkers in multifocal papillary thyroid cancer
Source: Cancer Cell Int. 2022 May 12;22:185. doi: 10.1186/s12935-022-02606-x (PMC9097102; doi:10.1186/s12935-022-02606-x)

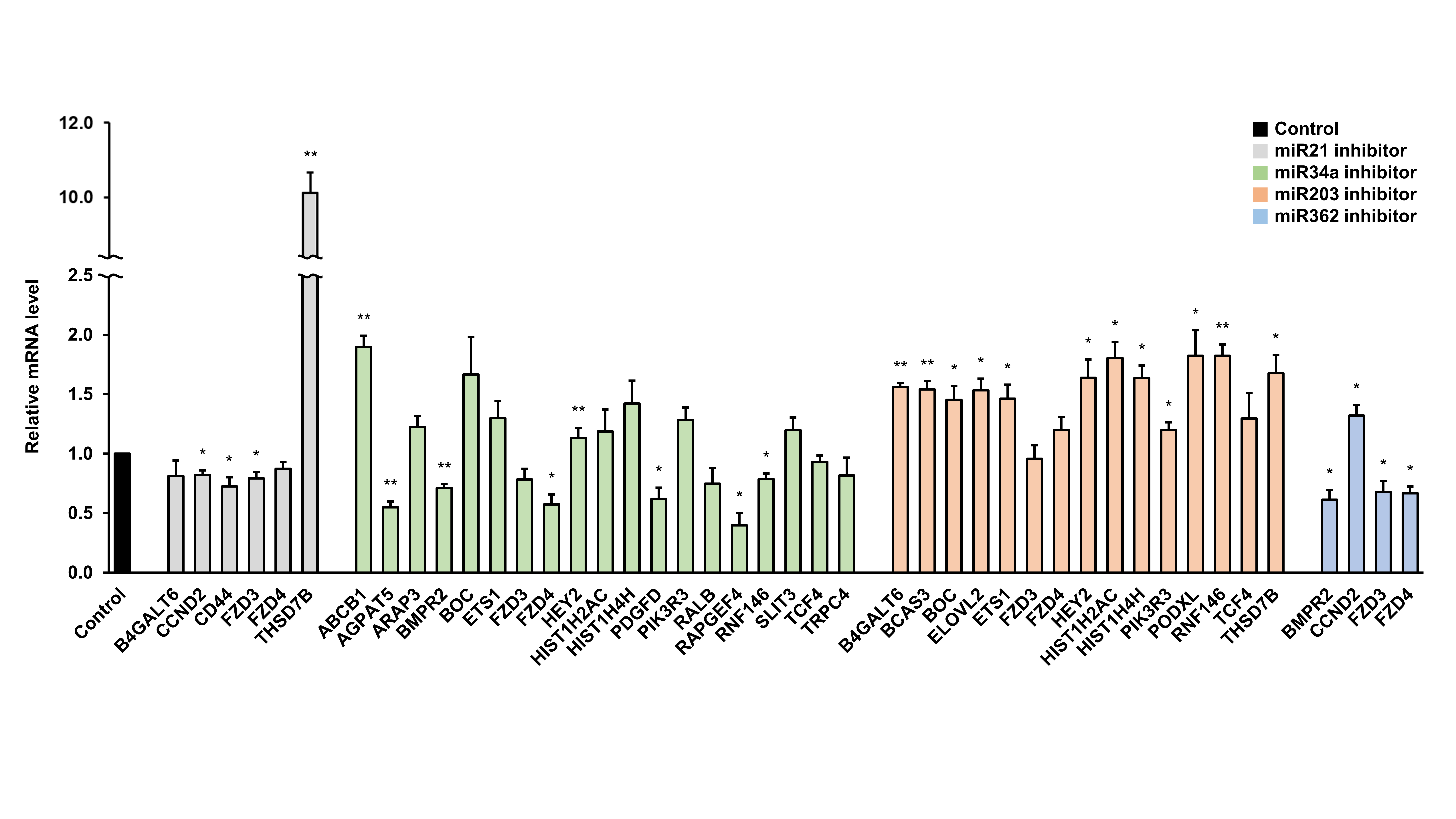

Supplement: Supplementary file 1 — Additional file 1: Figure S1. Expression changes of mRNAs in BRAF (+) BCPAP cells after treatment with miR21 (gray), miR34a (green), miR203 (orange), and miR362 (blue) inhibitors at 24hr. [file 12935_2022_2606_MOESM1_ESM.tif]

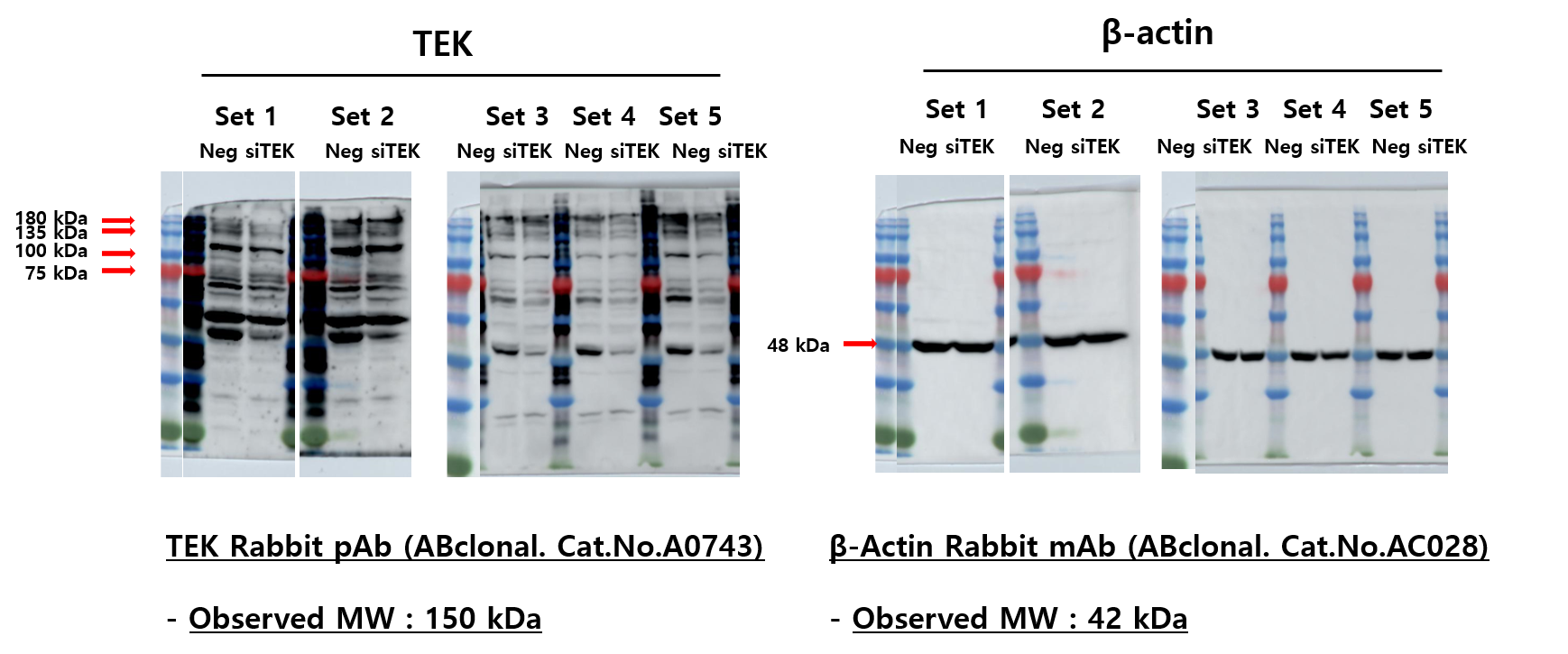

Supplement: Supplementary file 2 — Additional file 2: Figure S2. Raw Western blot data. [file 12935_2022_2606_MOESM2_ESM.tif]
